# Vitamin D Deficiency and the Risk of Recurrent Benign Paroxysmal Positional Vertigo

**DOI:** 10.7759/cureus.52433

**Published:** 2024-01-17

**Authors:** Taghreed S Saeed Al-Rawi, Raid M Al-Ani

**Affiliations:** 1 Department of Biochemistry, College of Medicine, University of Anbar, Ramadi, IRQ; 2 Department of Surgery, Otolaryngology, College of Medicine, University of Anbar, Ramadi, IRQ

**Keywords:** dizziness, serum calcium, vertigo, vitamin d, benign paroxysmal positional vertigo

## Abstract

Background

Benign paroxysmal positional vertigo (BPPV) is the most common cause of positional vertigo. It is a short-lived (seconds) rotatory attack of vertigo in relation to the position of the head. Vitamin D deficiency may be one of the causes leading to BPPV. As there is no relevant local study from Iraq, this study aimed to evaluate the association between BPPV and vitamin D deficiency.

Methodology

This retrospective, case-control study was conducted at the otolaryngology clinic of Al-Ramadi Teaching Hospital during a 26-month duration. The study included 40 patients clinically diagnosed with BPPV and 80 individuals as controls. Detailed information regarding the demographic and clinical characteristics was obtained from each participant. Serum vitamin D and calcium levels were measured for each participant.

Results

Both cases and controls were matched regarding age and gender. Serum vitamin D level in cases (15.458 ± 6.14 ng/mL) was lower than controls (23.604 ± 12.58 ng/mL), with a p-value of 0.0001 and large clinical effect size (0.8). Vitamin D deficiency was found in 35 cases and 37 controls, with a highly significant difference (p = 0.0001) and an odds ratio of 8.135. Vitamin D deficiency in BPPV patients with recurrence (12.615 ± 4.096 ng/mL) was lower than those without recurrence (18.3 ± 6.611 ng/mL), with a highly significant difference (p = 0.002) and small clinical effect size (0.3). Older age and vitamin D deficiency were risk factors for recurrence according to the multinominal logistic regression test (p < 0.05).

Conclusions

Vitamin D deficiency might cause the occurrence and recurrence of BPPV. Older age might be a risk factor for BPPV recurrence.

## Introduction

Benign paroxysmal positional vertigo (BPPV) is a widespread peripheral vestibular disease in otolaryngology clinics. Almost 10% of the population suffers from severe BPPV. BPPV is characterized by positional nystagmus or transient vertigo induced by the change of head in a particular critical position. It is mainly treated with rehabilitation therapy utilizing the canalith repositioning method. However, the recurrence rate of BPPV is approximately 50% [1]. The quality of life of BPPV patients is lower compared to the general population [2].

BPPV is generally caused by the displacement of otoconia from the utricle into the semicircular canals, leading to the degeneration of the vestibular system involving the otoliths. Otoconia is composed of glycoprotein and calcium carbonate crystals. Female gender, advanced age, hyperlipidemia, head trauma, hypertension, diabetes mellitus, migraine, cervical spondylosis, osteopenia/osteoporosis, otitis media, abnormal vestibular evoked myogenic potential, and long-term use of computers are risk factors for BPPV [3]. However, the exact etiology of otoconia dislodgement in idiopathic BPPV patients is not well known.

In the last decade, many studies have reported that vitamin D deficiency can be a risk factor for the occurrence or recurrence of BPPV [4,5].

Many studies have investigated the correlation between BPPV patients and low bone mineral density [6]. This is because the otoconia and bone might disintegrate in the same manner [7]. Bone formation and calcium homeostasis are directly related to the primary function of vitamin D. The correlation between serum vitamin D concentrations or calcium homeostasis and BPPV has been shown in several studies, demonstrating that decreased serum vitamin D concentrations are related to the recurrence of BPPV [8]. Several studies have assessed vitamin D supplements as a treatment modality in individuals suffering from BPPV. These studies illustrated that increasing serum vitamin D levels to the normal value can diminish BPPV recurrence [9]. However, several studies concluded no considerable relationship between vitamin D deficiency and BPPV [10].

Various factors can affect serum vitamin D levels, for example, gender, ethnicity, body weight, dietary characteristics, habitat, latitude, or seasons [11,12]. Therefore, there might be a variation in the concentration of serum vitamin D in the different study populations.

Furthermore, to our knowledge, the correlation between low vitamin D and BPPV has not been studied in Iraq. Moreover, geographical variations exist in the prevalence of vitamin D deficiency. Hence, this study was conducted to determine the correlation between low serum vitamin D concentrations and BPPV at the otolaryngology clinic of Al-Ramadi Teaching Hospital, Ramadi, Anbar, Iraq. The second objective was to determine if there is a difference in serum vitamin D levels in BPPV patients with single episodes versus recurrent attacks.

## Materials and methods

This retrospective, case-control study was conducted at the otolaryngology clinic of Al-Ramadi Teaching Hospital, Ramadi, Anbar, Iraq. The study was conducted from August 2021 to October 2023. The study was approved by the Ethical Approval Committee of the University of Anbar (reference number: 112 on 04-10-2023). Informed consent was waived owing to the retrospective nature of the study.

Patients with features suggestive of BPPV and approved clinically with positive Dix-Hallpike test or supine head roll test were enrolled in this study. Patients aged >18 years of both sexes were included in this study. Those taking vitamin D or calcium as a supplement for any cause, malignancy-related disease, parathyroid hormone-related disease, medication-related calcium metabolism, endocrine disorder, chronic kidney disease, inner ear surgery, a history of head injury, other causes of vertigo, aged ≤18 years, with incomplete records, or with a previous history of BPPV in the control group were excluded from the study.

We calculated the sample size for both groups using OpenEpi, version 3, at a 95% confidence interval, 80% power (% chance of detecting), a ratio of controls to cases of 2, and a hypothetical proportion of controls and cases with exposure of 41% and 70%, respectively, according to previous studies [12,13]. Accordingly, the sample size for controls and cases were 73 and 37, respectively. We approximated the sample size in our study to 80 for controls and 40 for cases for easy analysis.

Demographic (age and gender) and clinical (type of BPPV (posterior, superior, and lateral), side (left, right, or bilateral), and whether there were recurrent attacks or not) data were recorded for all participants. BPPV was considered to be recurrent when the individual experienced ≥two previous attacks of positional vertigo with the same presenting features, with an interval of ≥one month between each attack [14]. The age and gender of each participant in the control group were also recorded.

Venous blood was collected from participants to estimate total vitamin D (25-hydroxyvitamin D) concentration using fluorescence immunoassay technology (ichroma™ Vitamin D Neo IR2NL083056, Boditech Med Inc., Republic of Korea) and serum calcium level (reference range = 8.5-10.5 mg/dL) using the Semi-Auto Chemistry Analyzer/BA-88A (Biosystem, Spain). For the BPPV group, a blood test was done within a week after the first BPPV attack or recurrent BPPV. According to the results of serum vitamin D, participants were divided into the following two groups: deficient (<20 ng/mL) or not deficient (≥20 ng/mL).

The obtained data were entered and analyzed using SPSS version 22 (IBM Corp., Armonk, NY, USA). The quantitative variables, for example, age and serum vitamin D levels, were expressed as mean, median, range, and standard deviation (SD). The qualitative data, for example, gender and BPPV type (anterior, horizontal, and posterior), were expressed as proportions and percentages. We used Shapiro-Wilk and d’Agostino-Pearson tests to assess the normality of the continuous variables. The chi-square test was used to analyze categorical variables. We used the multivariate analysis of variance test to analyze two unbalanced samples. Multinominal logistic regression was performed to examine the impact of gender, age, serum vitamin D, and calcium levels on disease prediction (BPPV). The odds ratio was estimated with a 95% confidence interval. We calculated the clinical effect size using Cohen’s d equation (clinical effect size = (mean 1-mean 2)/SD). Values of 0.2-0.4 meant small, 0.5-0.7 moderate, and ≥0.8 large effects. P-values <0.05 were considered statistically significant.

## Results

The age of the patients with BPPV ranged from 21 to 75 years, with the majority of participants being female (n = 27, 67.5%). Most patients showed the left side (n = 18, 45%) and posterior semicircular canal involvement (n = 39, 97.5%). The number of patients with or without recurrence was equal (Table 1).

**Table 1:** Demographic and clinical characteristics of 40 patients with benign paroxysmal positional vertigo.

Variable	Frequency	Percentage
Age (year)		
Range	21–75	
Mean ± SD	48 ± 13.661	
Median	48	
Gender		
Female	27	67.5%
Male	13	32.5%
Side		
Left	18	45%
Right	10	25%
Bilateral	12	30%
Semicircular canal involved		
Posterior	39	97.5%
Lateral	0	0
Superior	1	2.5%
Recurrence		
Yes	20	50%
No	20	50%

There was no significant difference between cases and controls regarding age and gender (p = 0.385 and p = 0.78, respectively), as shown in Table 2.

**Table 2:** Comparison between the patient and control groups regarding age and gender.

Variable	Patient group (N = 40)	Control group (N = 80)	Total (N = 120)	P-value
Age (year)				0.385
Mean ± SD	48 ± 13.661	44.49 ± 12.792		
Gender				0.78
Male	13 (35.14)	24 (64.86)	37 (100%	
Female	27 (32.5)	56 (67.5)	83 (100%)	

There was a statistically significant difference (p = 0.0001) between the mean serum vitamin D level of both controls and cases, with a large clinical effect size (0.8). However, there was no statistically significant difference (p = 0.822) between the mean serum calcium level of controls and cases (Table 3).

**Table 3:** Comparison between the patient and control groups regarding the serum vitamin D and calcium levels.

Variable	Patient group (N = 40)	Control group (N = 80)	P-value	Clinical effect size
Vitamin D (ng/mL) (mean ± SD)	15.458 ± 6.14	23.604 ± 12.58	0.0001	0.8
Calcium (mg/dL) (mean ± SD)	8.96 ± 0.528	8.93 ± 0.552	0.822	-

The majority of cases had vitamin D deficiency (n = 35, 87.5%), and 46.25% of controls had vitamin D deficiency. There was a statistically significant difference (p = 0.0001) between both groups regarding vitamin D deficiency, with an odds ratio of 8.135 (Table 4).

**Table 4:** Association between serum vitamin D levels in patients with benign paroxysmal positional vertigo and controls.

Group	Vitamin D			Odds ratio	95% confidence interval		P-value
	Deficient, N (%)	Not deficient, N (%)	Total, N (%)		Lower	Upper	
Patient	35 (87.5)	5 (12.5)	40 (100)	8.135	2.890	22.901	0.0001
Control	37 (46.25)	43 (53.75)	80 (100)				
Total	72 (60)	48 (40)	120 (100)				

There was a statistically significant difference (p = 0.002) between the mean serum vitamin D levels of patients with or without recurrence (p = 0.002). However, the clinical effect size was small (0.3). However, there was no statistically significant difference (p = 0.703) between the mean serum calcium levels of patients with or without recurrence (Table 5).

**Table 5:** Comparison between patients with the first attack or recurrent attacks regarding the mean serum vitamin D and calcium levels.

Variable	First attack (N = 20)	Recurrent attacks (N = 20)	P-value	Clinical effect size
Vitamin D (ng/mL) (mean ± SD)	18.3 ± 6.611	12.615 ± 4.096	0.002	0.3
Calcium (mg/dL) (mean ± SD)	8.93 ± 0.614	8.99 ± 0.439	0.703	-

Older age and vitamin D deficiency were risk factors for recurrence in BPPV patients (p < 0.05), as shown in Table 6.

**Table 6:** Factors affecting benign paroxysmal positional vertigo recurrence using multinomial logistic regression test.

Variable	P-value
Age (year)	0.0286
Gender	0.9079
Vitamin D (ng/mL)	0.0109
Calcium (mg/dL)	0.4104

## Discussion

BPPV is one of the most common abnormalities of the peripheral vestibular system seen in neurology or otolaryngology clinics. BPPV has a lifetime prevalence of 2.4% with a wide age range of onset (11 to 84 years), comprising 20-30% of vestibular vertigo [15]. Although many studies have investigated the possibility of vitamin D deficiency as a cause of BPPV or its recurrence, there is controversy regarding this issue [14,16,17]. Furthermore, several factors might affect the serum vitamin D levels such as gender, ethnicity, body mass index, dietary habits, latitude, or seasonal variation [11,12]. As there is no relevant research from Iraq to our knowledge, this study was conducted. The main outcome of this study was that vitamin D deficiency can be a cause of BPPV or its recurrence.

Our finding was consistent with several studies in that BPPV is more prevalent in females [16,18,19]. This may be attributed to the low estrogen levels that result in reduced bone regulation, leading to changes in the internal otoconia components and their relation to the gelatinous matrix [18].

The mean age of our patients with BPPV (48 ± 13.661 years) was lower compared to other studies [5,10,16,19]. However, it was nearly similar to two other studies from Egypt [4,18]. This might be due to geographical differences among various studies.

In this study, the posterior semicircular canal was the most canal involved in patients with BPPV [19]. Contrary to the belief that BPPV has a predilection for the right posterior semicircular canal [19,20], in this study, the left side involvement was more predominant than the right side or bilateral involvement. The authors did not find a cause for this contradiction.

Vitamin D deficiency is more prevalent in patients with BPPV than in the general population. A recent study from China reported that 62.1% and 42.8% had low serum levels of vitamin D in BPPV cases and controls, respectively [14]. Shin et al. found that low serum vitamin D level is a risk factor for BPPV recurrence [21]. A study from India reported that a statistically significant difference (p = 0.001) between idiopathic BPPV and low vitamin D concentration [22]. Pecci et al. from Italy suggested a relationship between vitamin D deficiency and BPPV onset. Moreover, they reported that the correction of hypovitaminosis can reduce both the number of recurrent attacks and the number of episodes per patient and that there is a significant effect of vitamin D supplementation regarding the responsiveness of BPPV to physical therapy [23]. Our results were consistent with the above-mentioned studies in that low serum vitamin D might be a cause of BPPV or its recurrence. On the contrary, Işık et al. did not find a relationship between vitamin D and total calcium serum levels and BPPV prevalence or recurrence [17]. A study from Croatia reported that low vitamin D3 serum level is not a risk factor for BPPV recurrence [10]. Another study from India concluded that there is no association between calcium and vitamin D concentrations and BPPV [24]. A recent investigation from the United States found that BPPV patients had a significantly higher vitamin D level than the NHANES (National Health and Nutrition Examination Survey) individuals (31.4 ±16.5 vs. 26.0 ±11.2 ng/mL, d = 0.474 (0.323, 0.626)) [16]. Furthermore, BPPV patients with recurrences had significantly lower vitamin D serum levels at initial presentation when compared to patients with no recurrences (29.0 ±12.0 vs. 37.6 ±18.3 ng/mL, d = 0.571 (0.139,1.001)) [16]. The differences among different studies might be attributed to the differences in the clinical settings. In addition, serum vitamin D concentrations were also affected by different factors, such as gender, age, seasonal effects, hormonal effects, geographical location, nutrition and lifestyle habits, and metabolic diseases [11,12].

Although this study found that vitamin D deficiency was significantly higher in cases than in controls, such an association was not reported (p < 0.05) between the serum calcium level in BPPV patients and controls or BPPV patients with or without recurrence.

Owing to geographical differences regarding vitamin D deficiency, this study has a limitation, i.e., it was a single-center study. The retrospective nature of the study is another limitation. Although the present study compared the means of serum vitamin D levels between the groups, the division of BPPV patients and controls into two groups, irrespective of deficient serum vitamin D levels, could have affected the accuracy of the results, which is another limitation of this study.

## Conclusions

This study reported a significant association between cases and controls regarding vitamin D deficiency. Moreover, there was a significant association between BPPV patients with or without recurrence regarding vitamin D deficiency. This suggests that vitamin D deficiency might be a risk factor for BPPV occurrence or its recurrence. Additionally, older age could be a risk factor for BPPV recurrence. However, we recommend multicenter studies to verify the relationship between BPPV and vitamin D deficiency.
